# Editorial

**Published:** 1853-06

**Authors:** 


### THE MEDICAL EXAMINER.

PHILADELPHIA, JUNE, 1853.

A considerable portion of our space in this number of the Examiner is occupied with Reports of the Proceedings of the American Medical Association and of our State Medical Society, both of which held their annual sessions within the past month. We have no doubt that the detailed accounts of their Proceedings which are presented to our readers will be acceptable, for we are satisfied that the interest which is felt in the working of our professional organization is strong and universal. Our reports of both the National and State Proceedings will be found, we think, a full and faithful record of everything of any interest that transpired at either meeting. In the preparation of the Report of the Proceedings of the American Medical Association, we have had the opportunity of completing our own notes from the excellent reports of the New York Daily and Medical Times, and of the New Jersey Medical Reporter, to all of which our acknowledgments are due. The Report of the Proceedings of the State Medical Society was prepared from notes taken for the Examiner.

It was the privilege of the editors of this Journal to be present at the late meeting of the American Medical Association at New York, and we are indeed at a loss for language to express our sense of the magnificent public reception and cordial private hospitality which the delegates to that body received from their professional brethren in New York. We can only say that it was a reception worthy of the great metropolis of the nation and of her high-toned physicians.

The transactions of the Association were characterized by the greatest harmony and discretion, and in interest and importance the scientific reports and other papers presented and read were not inferior to those of previous meetings. As regards the proposed amendments to the Constitution, which came up from last year's meeting, they were indefinitely postponed, with the exception of the amendment in favor of a representation from the Medical Staffs of the Army and Navy.

The Proceedings of our State Medical Society were characterized by the zeal and spirit which they have always elicited. Eighty-five members were in attendance, and a very large number of highly valuable reports from the County Medical Societies were presented and read. The meeting was in every respect a most satisfactory and agreeable one.

